# Zinc is essential for the transcription function of Nrf2 in human renal tubule cells *in vitro* and mouse kidney *in vivo* under the diabetic condition

**DOI:** 10.1111/jcmm.12239

**Published:** 2014-03-06

**Authors:** Bing Li, Wenpeng Cui, Yi Tan, Ping Luo, Qiang Chen, Chi Zhang, Wei Qu, Lining Miao, Lu Cai

**Affiliations:** a Department of Nephrology, The Second Hospital of Jilin UniversityChangchun, China; b Kosair Children*s Hospital Research Institute, and Departments of Pediatrics and Pharmacology and Toxicology, University of LouisvilleLouisville, KY, USA; c Department of Nephrology, Jilin Province People*s HospitalChangchun, China; d Chinese-American Research Institute for Diabetic Complications, Wenzhou Medical UniversityWenzhou, China; e Inorganic Toxicology Group, National Toxicology Program Laboratory, Division at the National Toxicology Program, National Institute of Environmental Health SciencesResearch Triangle Park, NC, USA; f Department of Radiation Oncology, The University of LouisvilleLouisville, KY, USA

**Keywords:** Akt phosphorylation, diabetic nephropathy, Fyn, Nrf2, TPEN, zinc chelation

## Abstract

Increasing evidence from human and laboratory studies showed the effect of zinc (Zn) on diabetic complications. Nuclear factor-erythroid 2-related factor 2 (Nrf2) plays important role in the prevention of oxidative damage. This study was to define whether Zn statues (deficiency or supplement) affect the Nrf2 expression and function, and also affect the damage severity of human renal tubular (HK11) cells exposed to high glucose (HG) with palmitate (Pal) and kidney of diabetic mice induced by multiple low-dose streptozotocins. For Zn deficiency diabetic mice were treated with Zn chelator PTEN at 5 mg/kg bw daily for 4 months. Results showed that HG/Pal significantly increased the expression of pro-fibrotic mediators, connective tissue growth factor and PAI-1, in HK11 cells, which was exacerbated by TPEN that depleted intracellular free Zn and decreased Nrf2 expression and transcription. Zn supplement prevented the effects of TPEN and also increased Akt and GSK-3β phosphorylation with a decrease in Nrf2 nuclear exporter, Fyn. All these effects of Zn were abolished by Akt inhibitor. Therefore, Zn up-regulates Nrf2 function *via* activating Akt-mediated inhibition of Fyn function. Treatment of diabetic mice with TPEN decreased renal Zn level and Nrf2 expression and transcription, with an exacerbation of renal oxidative damage, inflammation and fibrosis. These results suggest the essentiality of Zn for Nrf2 expression and transcription function.

## Introduction

Zinc (Zn) is an essential trace metal for eukaryotes, with numerous physiological functions and also acts as a cofactor for many enzymes and proteins [1]. Zn restriction during different periods of life is associated with an increase in renal and cardiovascular diseases [2,3]. Recently, the importance of Zn in the development of diabetes and its cardiovascular diseases has been obtaining attention. Meta-analyses indicated the beneficial effect of Zn supplementation on glycemic control in diabetic patients [4,5]. High risk of cardiovascular events was also found in the diabetic patients with low serum Zn levels [6]. In animal models, we have shown that Zn deficiency significantly increased diabetes-induced damage in different organs [7–9]. In contrast, Zn supplementation in diabetic mice and rats significantly reduces diabetes-induced cardiac and renal pathological damage [10,11], which was phenomenally confirmed by recent two studies [12,13].

The above human and animal models suggest the importance of Zn status in the development of diabetic complications. For Zn protection against diabetes-induced organ damage, induction of the potent antioxidant metallothionein (MT) has been proposed as one of the underlying mechanisms [10,11]. However, the finding by Apostolova *et al*. that Zn*s protection from streptozotocin (STZ)-induced damage is independent of MT [14] suggests that at least other components also play important roles in mediating the protective role of Zn in diabetic damage. Reportedly, the antioxidant effect of Zn may be mediated by the up-regulation of nuclear factor-erythroid 2-related factor 2 (Nrf2) [15,16].

Nuclear factor-erythroid 2-related factor 2 is one of the most important cellular defense factors against oxidative stress [17]. It regulates intracellular antioxidants and other proteins to neutralize reactive oxygen and/or nitrogen species (ROS and/or RNS). NAD(P)H quinone oxidoreductase-1 (NQO-1), heme oxygenase-1 (HO-1), superoxide dismutase (SOD) and glutathione S-transferase are among the well-studied Nrf2 target genes [17,18]. As an adaptive mechanism, Nrf2 is quickly up-regulated in cells and tissues in response to oxidative stress at early stage, but down-regulated in cells or tissues exposed to chronic or long-lasting oxidative stress [19,20]. A few studies have indicated the induction of Nrf2 expression and function by Zn [15,16,21]. Therefore, it has been suggested that the exacerbation of diabetes-induced organ damage by Zn deficiency may be because of the down-regulation of Nrf2 expression and function.

Diabetic nephropathy has become one of the leading causes of death in diabetic patients and is related to oxidative stress as a result of extra generation of ROS or RNS in the kidney [22]. The important role of Nrf2 in preventing diabetes-induced oxidative stress was also established recently. First, Nrf2 was quickly up-regulated in tissues or cells in response to hyperglycaemia or high glucose (HG) [17,18]. Second, diabetes-induced cardiac and renal damage in Nrf2 gene knockout (Nrf2-KO) mice was more severe than that in wild-type (WT) mice [17,23,24]. Third, activation of Nrf2 by sulforaphane (SFN) *in vitro* and *in vivo* or MG132 *in vivo* suppressed HG-induced ROS and metabolic dysfunction in human microvascular endothelial cells [25] and attenuated diabetic proteinuria in STZ-induced diabetic rats [26,27]. Sulforaphane treatment that can activate renal Nrf2 function showed renal protection only in WT mice, but not in Nrf2-KO mice [27]. Therefore, if Zn deficiency impairs the expression and function of Nrf2, it will exacerbate diabetes-induced pathogenic process in the kidney.

In this study, therefore, using human renal tubule HK 11 cells we first investigated the direct effects of Zn status on renal damage induced by an *in vitro* diabetes-mimic condition: HG plus high lipid (palmitate, Pal). Second, we also examined whether Zn deficiency and supplement affects Nrf2 expression and function. Then animal models were used to confirm the *in vitro* findings, for which a mild Zn deficiency was induced in type 1 diabetic mouse model. Diabetes was induced with multiple low-dose STZ (MLD-STZ) and mild Zn deficiency was induced by chronic treatment with Zn chelator, N,N,N′,N′-tetrakis (2-pyridylemethyl) ethylenediamine (TPEN) for 4 months, as used by others [28,29].

## Materials and methods

### Cell culture and treatments

HK11 cells were maintained in DMEM/F12 supplemented with 5% foetal bovine serum. Cells were exposed either to D-glucose in a final concentration of 27.5 mM (HG) or to 5.5 mM D-glucose plus 22 mM D-mannitol as hyperosmotic control for 24 hrs. To mimic diabetic *in vivo* conditions that not only include hyperglycaemia, but also include hyperlipidaemia, the culture was added with both HG and palmitate, as described before [10]. Therefore, the culture medium was replaced by 2% bovine serum albumin for another 24 hrs with HG, in which palmitate (300 μM) was added at the last 6 hrs. To make Zn deficiency in cultured cells, TPEN at 4–8 μM was added into the medium of some cultures for 30 hrs.

### Type 1 diabetic mouse model and Zn depletion

All animal procedures were approved by the Institutional Animal Care and Use Committee, which is certified by the American Association for Accreditation of Laboratory Animal Care. FVB mice at 8 weeks of age were injected intraperitoneally with MLD-STZ (Sigma-Aldrich, St. Louis, MO, USA) at 50 mg/kg daily for 5 days, as described previously [18,30]. Five days after the last injection of STZ, whole-blood glucose obtained from mouse tail-vein was measured with a SureStep complete blood glucose monitor. Blood glucose levels higher than 250 mg/dl were considered as hyperglycaemic. Diabetic and age-matched control mice were treated intraperitoneally with TPEN (Sigma-Aldrich) at 5 mg/kg daily or vehicle for 4 months. The selection of TPEN to chronically deplete systemic Zn is based on several previous studies that have successfully used TPEN to mildly lower the Zn levels *in vivo* and intracellular free Zn *in vitro* without significant systemic toxic effects [28,29].

### Measurement of renal Zn level

Renal Zn level was measured by an atomic absorption spectrophotometer using air-acetylene flame after tissue digestion by nitric acid, as described previously [31].

### Histopathological and immunofluorescent examination

Kidneys were collected for processing the fixation, embedding and sectioning for periodic acid-Schiff (PAS) staining, as reported previously [32]. Based on PAS staining, pathological changes of glomeruli were examined under light microscope. Tissue sections were also stained with 0.1% Sirius-red F3BA and 0.25% Fast green FCF, to detect the proportion of fibrosis (collagen) in the kidney tissues using computer image analysis system [32].

For renal inflammatory cell infiltration, Naphthol AS-D Chloroacetate esterase kit was used to stain for the specific esterase of leucocytes, mastocytes and some macrophages, based on the instruction provided by the kit (Sigma-Aldrich).

Immunofluorescent stain was also used specifically with rabbit monoclonal anti-collagen IV antibody (Santa Cruz Biotechnology, Santa Cruz, CA, USA). Semi-quantitative analysis was blindly performed for the percentage of positive staining cells using computer imaging system.

### Western blotting assays

Kidney tissues were homogenized in lysis buffer and the supernatant were collected by centrifuging at 12,000 × g at 4°C for 10 min. For cultured HK11 cells, monolayer cultured cells were collected with a rubber cell scraper and lysed by RIPA buffer (Santa Cruz Biotechnology). Proteins from tissues or cells, diluted in loading buffer and heated at 95°C for 5 min., were separated electrophoretically with 10% SDS-PAGE gel at 120 V and transferred to nitrocellulose membrane. After proper wash and blocking for the non-specific antigen, membranes were incubated with different primary antibodies overnight at 4°C and then reacted with secondary horseradish peroxidase–conjugated antibody, as stated earlier [32]. Antigen-antibody complexes were visualized using ECL kit (Amersham, Piscataway, NJ, USA). After detection of the primary target proteins, membranes were stripped and reprobed with monoclonal anti-actin antibody as a loading control [18,30].

Antibodies against intracellular adhesion molecule-1 (ICAM-1; 1:500), transforming growth factor (1:500), connective tissue growth factor (CTGF; 1:500) and Zn-copper SOD (SOD1; 1:2000) were purchased from Santa Cruz Biotechnology. Antibodies against HO-1 (1:500), total and phosphorylated Akt (1:1000 and 1:500), total and phosphorylated GSK-3β (1:1000 and 1:500), and Fyn (1:1000) were purchased from Cell Signaling Technology (Danvers, MA, USA). The primary antibodies also included those against plasminogen activator inhibitor type 1 (PAI-1, 1:2000; BD Biosciences, Sparks, MD, USA), Nrf2 (1:000; Abcam, Cambridge, MA, USA), 3-nitrotyrosine (3-NT, 1:1000; Chemicon, Billerica, CA, USA) and 4-hydroxynonenal (4-HNE, 1:2000; Calbiochem, San Diego, CA, USA).

### Visualization of Nrf2 nuclear accumulation by fluorescent microscope

Nuclear factor-erythroid 2-related factor 2 nuclear translocation was monitored by fluorescent microscope. We cultured HK11 cells on chamber slides that were then incubated with rabbit anti-Nrf2 antibody (1:50) overnight at 4°C, with Cy3–conjugated goat anti-rabbit antibodies and stained with Dapi for the nuclei. Cellular free Zn levels were detected by fluorescent probe Zinquin (Invitrogen Co., Grand Island, NY, USA), as used by others [33].

### Statistical analysis

Data were collected from repeated experiments and presented as mean ± SD. One-way anova was used to determine if differences exist and if so, a *post hoc* Tukey*s test was used for analysis for the difference between groups, with Origin 7.5 laboratory data analysis and graphing software. Statistical significance was considered as *P* < 0.05.

## Results

### Effects of Zn statuses on HG and/or Pal-induced expression of PAI-1, CTGF and Nrf2 in the cultured human renal tubular cells

To explore whether Zn deficiency increases diabetes-induced damage, and if so, what is the potential mechanisms, human renal tubular HK11 cells were used to model the *in vivo* renal fibrotic effect and investigate the direct effect of Zn deficiency and supplement on HG/Pal-induced fibrotic effects, expression of PAI-1 and CTGF.

**Fig. 1:**
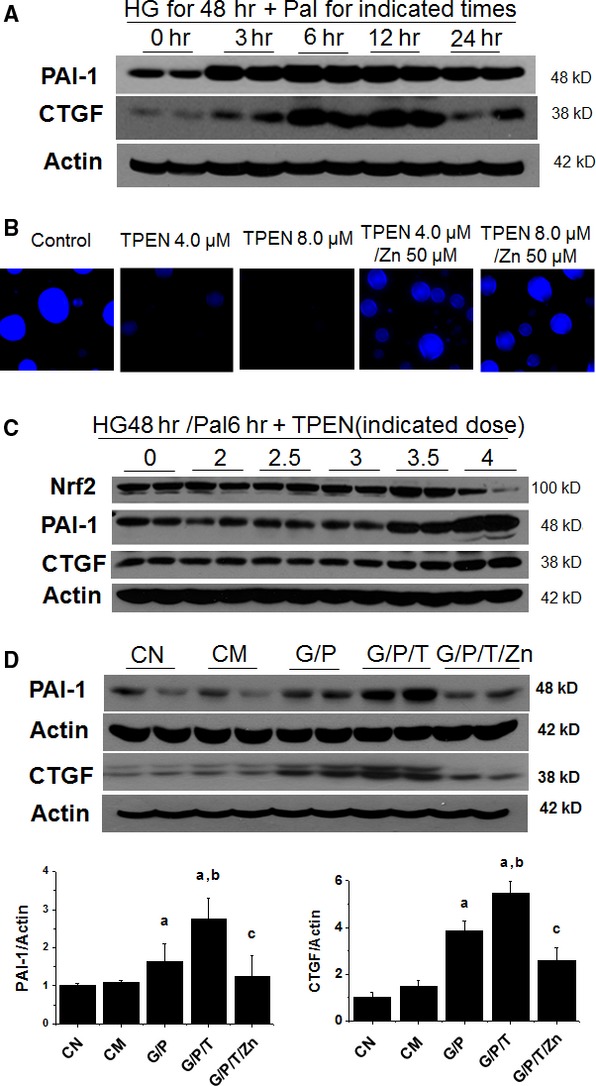
Effect of TPEN on intracellular free Zn levels, Nrf2, PAI-1 and connective tissue growth factor (CTGF) expression of the cultured human renal tubular cells. Human renal tubular HK11 cells were cultured on chamber slides for fluorescent staining and in flasks for Western blotting assays. Cells were treated with HG (27.5 mM) for 48 hrs and Pal (P, 300 μM) for the last 6 hrs and then cellular expression of PAI-1 and CTGF was examined by Western blotting (**A**); Cells were treated with TPEN at 4.0 or 8.0 μM with and without Zn at 50 μM for 30 hrs, and then subject to fluorescent staining with Zinquin (**B**). Cells were treated with HG for 48 hrs and Pal for the last 6 hrs with different concentrations of TPEN [0 (T0), 2 (T2), 2.5 (T2.5), 3 (T3), 3.5 (T3.5) and 4.0 (T4) μM] for 30 hrs and then the protein expression of Nrf2, PAI-1 and CTGF were detected by Western blotting assays (**C**); Cells were treated with HG for 48 hrs, Pal for the last 6 hrs, TPEN (T, 4 μM) with or without Zn (50 μM) for the last 30 hrs and then were subject to Western blotting assay for the expression of PAI-1 and CTGF (**D**). Experiments were repeated at least three times and the data are presented as the mean ± SD. a, *P* < 0.05 *versus* CN or CM group; b, *P* < 0.05 *versus* G/P group; c, *P* < 0.05 *versus* G/P/T group. CN: control; CM: hyperosmotic control; G: HG; P: Pal; T: TPEN.

First, HK11 cells were exposed to HG (27.5 mM) and/or Pal (300 μM). We found that HG induced PAI-1 from 3 to 48 hrs (data not shown), as reported before [34]. We selected the treatment with HG for 48 hrs to further explore the effect of HG plus Pal from 3 to 24 hrs. HG/Pal increased the expression of PAI-1 and CTGF (Fig. 1A) from 3 to 12 hrs with a peak at 6 hrs. Therefore, in the following experiments, we treated HK11 cells with HG for 48 hrs and Pal for the last 6 hrs with or without other treatments.

Second, HK11 cells were exposed to TPEN at different doses (4.0–8.0 μM) to ensure reduction in cellular free Zn by TPEN [33]. Fluorescent staining for Zn by Zinquin revealed the intense fluorescence in normal cells and significantly less fluorescence in the cells treated with either 4.0 or 8.0 μM TPEN for 30 hrs (Fig. 1B). The intense fluorescent staining was evident again when TPEN-treated cells were supplemented with Zn (50 μM), suggesting that the addition of extra exogenous Zn can overcome the reductive effect of TPEN on cellular free Zn.

For the third study, we treated HK11 cells with HG for 48 hrs and Pal for the last 6 hrs with different doses of TPEN for the last 30 hrs. Compared to HG/Pal treatment alone (0 group), HG/Pal with TPEN at 4 μM (4 group) significantly increased PAI-1 and CTGF expression, but decreased Nrf2 expression (Fig. 1C). Figure 1D shows that the enhancement of PAI-1 and CTGF expression by TPEN in HG/Pal (G/P/T) group was abolished by addition of Zn at 50 μM (G/P/T/Zn group). These results suggest that Zn deficiency or supplement decreases or enhances Nrf2 expression, respectively, in renal tubule cells under an *in vitro* diabetic condition (HG/Pal).

**Fig. 2:**
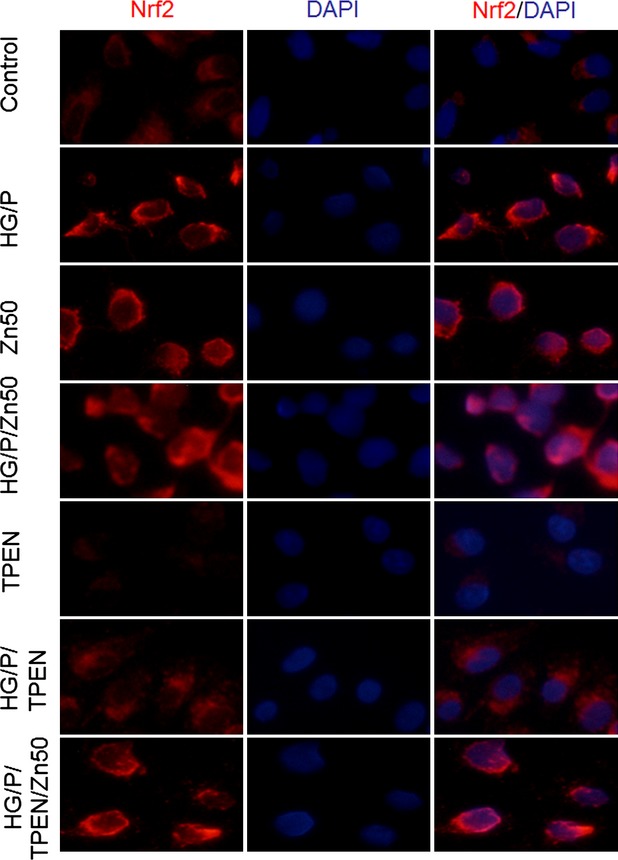
Nrf2 expression and nuclear translocation under different conditions. HK11 cells cultured on chamber slides were treated with different conditions as indicated: HG for 48 hrs, Pal for the last 6 hrs, TPEN (8 μM) and Zn (50 μM) for the last 30 hrs. Immunofluorescent staining of Nrf2 is detected by fluorescent microscope (400 × ). DAPI dying was used for nuclear staining.

Next, whether Zn affects Nrf2 expression in cellular compartments was examined by immunofluorescent staining for Nrf2. Treatment with either HG or Pal alone did not significantly affect Nrf2 expression that was predominantly localized in the cytosol (Fig. S1). However, treatment with HG/Pal increased Nrf2 expression predominantly in the cytosol (Fig. 2). Treatment with Zn at 50 μM significantly increased nuclear expression of Nrf2. Exposure of cells to Pal, but not HG, in the presence of Zn significantly increased nuclear expression of Nrf2 (Fig. S1). Zn treatment of cells that were exposed both HG/Pal also significantly increased the nuclear Nrf2 expression (HG/Pal/Zn, Fig. 2). If cells were treated with TPEN, Nrf2 expression was significantly down-regulated even in cytosol (Fig. 2). Compared to cells exposed to HG/Pal alone (HG/Pal/TPEN, Fig. 2), cells exposed to HG/Pal with TPEN showed a further down-regulation of Nrf2 expression, an effect that was completely abolished by Zn treatment under the same conditions (HG/Pal/TPEN/Zn, Fig. 2).

**Fig. 3:**
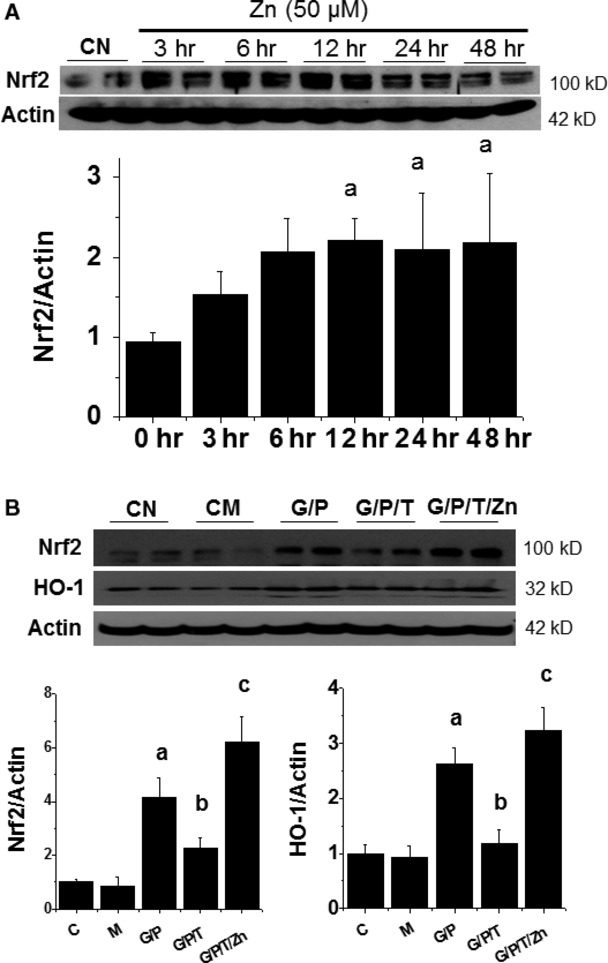
Zn is required for Nrf2 expression and function. HK11 cells were treated by Zn at 50 μM for indicated times and then Nrf2 expression was examined by Western blotting assays (**A**). HK11 cells were treated by HG (G, 27.5 mM) for 48 hrs, Pal (P, 300 μM) for the last 6 hrs and TPEN (T, 8 μM) with or without Zn (50 μM) for the last 30 hrs, and then subject to Western blotting for Nrf2 expression and its downstream gene HO-1 expression (**B**). Experiments were repeated at least three times and the data are presented as the mean ± SD. a, *P* < 0.05 *versus* CN or CM group; b, *P* < 0.05 *versus* G/P group; c, *P* < 0.05 *versus* G/P/T group. CN: control; CM: hyperosmotic control; G: HG; P: Pal; T: TPEN.

To determine the dynamic effect of Zn on Nrf2 expression, HK11 cells were treated with Zn at 50 μM for 3–48 hrs. Nrf2 expression was significantly up-regulated from 6 to 48 hrs (Fig. 3A). To define the effect of Zn levels on the restoration of TPEN-down-regulated Nrf2 expression and function, we administered Zn at 0, 25, 50, 75 and 100 μM for 30 hrs to the cells treated with HG/Pal plus TPEN at 4 μM. Zn induced a dose-dependent expression and nuclear localization of Nrf2, compared to the cells treated with TPEN alone (Figs. S2 and S3), suggesting that Zn is able to restore Nrf2 expression and nuclear translocation that was down-regulated by TPEN.

To further ensure the functional restoration of Nrf2 by Zn in the cells exposed to HG/Pal/TPEN, the expression of Nrf2 and its effector HO-1 was examined by Western blotting (Fig. 3B). HG/Pal slightly increased both Nrf2 and HO-1 expression. TPEN treatment with HG/Pal significantly down-regulated Nrf2 and HO-1 expression while Zn treatment at 50 μM completely restored the expression Nrf2 and HO-1 in the cells treated with HG/Pal/TPEN, suggesting the requirement of Zn for Nrf2 expression and transcription.

To confirm that Zn requirement for Nrf2 expression and function is a general feature, we further investigated the effect of Zn deficiency and supplement on the Nrf2 expression and function in response to SFN, an well-known Nrf2 inducer [35,36], which confirmed the suppression or enhancement of SFN-induced Nrf2 expression and function (measured by NQO-1) with Zn deficiency or Zn supplement (Fig. S4).

### Zn induced Nrf2 up-regulation *via* Akt-mediated prevention of Fyn nuclear translocation

**Fig. 4:**
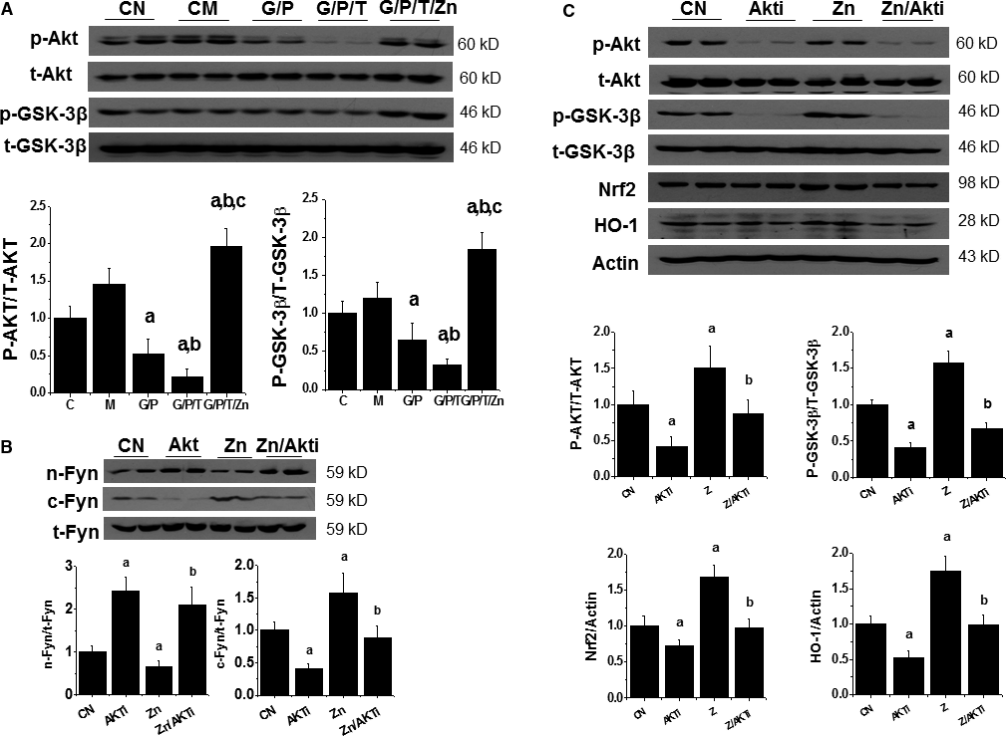
Zn-stimulated Nrf2 expression and function is associated with reduction in Fyn nuclear accumulation that is Akt dependent. HK11 cells were treated by HG (G, 27.5 mM) for 48 hrs, Pal (P, 300 μM) for the last 6 hrs and TPEN (T, 8 μM) with or without Zn (50 μM) for the last 30 hrs, and then subject to measurement for the expression and phosphorylation of Akt and GSK-3β (**A**). HK11 cells were treated with Zn (50 μM) with or without PI3K inhibitor, LY294002 (10 μM) for 24 hrs. Total Fyn expression along with nuclear and cytosolic accumulation of Fyn was examined by Western blotting (**B**). Under the (**B**) condition, expression and phosphorylation of Akt and GSK-3β and also the expression of Nrf2 and HO-1 were examined with Western blotting (**C**). Experiments were repeated at least three times. Data are presented as mean ± SD. a, *P* < 0.05 *versus* CN group; b, *P* < 0.05 *versus* Zn group. CN: control; Akti: PI3K inhibitor, Ly294002.

To explore mechanism by which Zn stimulates Nrf2 transcription function, we investigated the effect of Zn on Akt and GSK-3β phosphorylation as Zn was reported to stimulate their phosphorylation [1,37]. Figure 4A shows that HG/Pal decreased, and TPEN further decreased, Akt and GSK-3β phosphorylation, which was restored or even further stimulated by Zn addition.

**Fig. 5:**
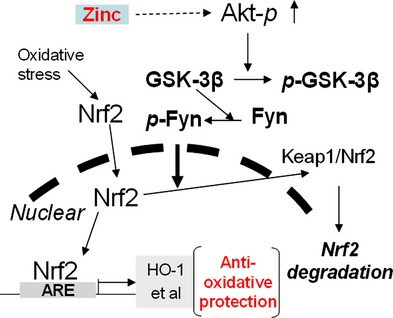
A proposed mechanism by which Zn up-regulates Nrf2 expression and function is illustrated.

Fyn is a well-known Nrf2 negative regulator by entering into nuclei to export Nrf2 to the cytosol where Nrf2 is degraded [38]. The translocation of Fyn into the nucleus is mediated by GSK-3β that is active in dephosphorylated form and inactive in phosphorylated form [39]. It has been suggested that Zn stimulation of Akt phosphorylation and, consequently, GSK-3β phosphorylation, may result in the reduction in Fyn nuclear translocation to export Nrf2 to the cytosol. HK11 cells were exposed to Zn with or without Akt inhibitor and then their nuclear and cytosol proteins were separated, which shows that Zn induced a significant decrease in Fyn expression in nuclear and an increase in cytosolic (Fig. 4B), along with the up-regulation of Akt and GSK-3β phosphorylation, and Nrf2 and HO-1 expression (Fig. 4C). Stimulating effects of Zn on Nrf2 and HO-1 expression (Fig. 4C) and Fyn cytosolic translocation (Fig. 4B) were all significantly prevented by Akt inhibitor through the inhibition of Akt and GSK-3β phosphorylation (Fig. 4B), which was mechanistically illustrated in Figure 5.

### Zn deficiency enhanced diabetes-induced renal oxidative damage, inflammation and fibrosis

Zn deficiency was induced by chronic TPEN treatment in MLD-STZ-induced diabetic mice as described in previous studies [7–9]. Chronic treatment with TPEN did not affect blood glucose level either in non-diabetic or diabetic mice (Fig. S5A). The ratio of kidney weight to bw was significantly increased in diabetes and diabetes/TPEN groups compared to control or TPEN group (Fig. S5B). Compared to control and diabetes mice, chronic TPEN treatment significantly decreased renal Zn level either in normal or diabetes mice (*P* < 0.01, Fig. S5C).

**Fig. 6:**
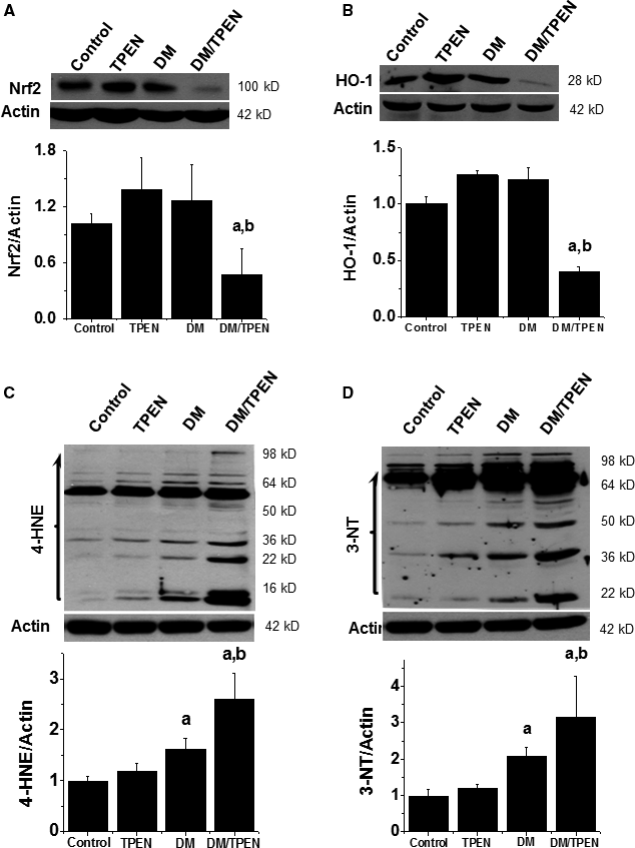
Effects of Zn deficiency on renal Nrf2 expression and function as well as nitrosative and oxidative damage. Renal tissues were subjected to Western blotting of Nrf2 expression (**A**), Nrf2-downstream target gene HO-1 expression (**B**), 4-hydroxynonenal (4-HNE, **C**) and 3-nitrotyrosine (3-NT, **D**) accumulation. Data are presented as mean ± SD (*n* = 5 at least in each group). a, *P* < 0.05 *versus* Control group; b, *P* < 0.05 *versus* DM group. DM: diabetes.

Analysis of renal Nrf2 expression revealed that TPEN treatment (Zn deficiency) or diabetes had slightly increased renal Nrf2 expression compared to control (*P* > 0.05, Fig. 6A). Zn deficiency was associated with a significant reduction in renal Nrf2 expression in Diabetes/TPEN group (*P* < 0.05, Fig. 6A). A similar pattern of the expression of HO-1 as one of Nrf2 effectors was found (Fig. 6B).

Zn deficiency in normal mice did not significantly increase renal oxidative and nitrosative damage, measured by lipid peroxidation with 4-HNE as an index of oxidative damage (Fig. 6C), and protein nitration with 3-NT as an index of nitrosative damage (Fig. 6D). Diabetes induced significant increase in 4-HNE and 3-NT in multiple proteins from 16 to 64 kD, which was significantly enhanced by Zn deficiency (Fig. 6C and D).

**Fig. 7:**
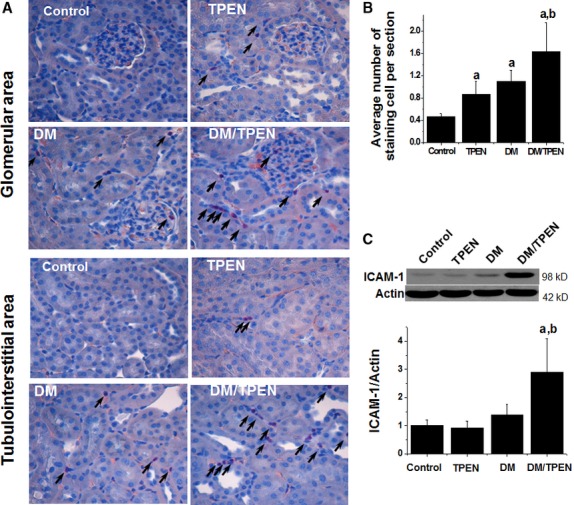
Effects of Zn deficiency on the renal infiltration of inflammatory cells and renal inflammatory. Inflammatory cells were examined by Naphthol AS-D Chloroacetate esterase staining (**A**). The images of this staining from each group are representatively provided for glomeruli and tubulointerstitial areas (original magnification ×400). Arrows indicate the positive cells. Positive cells for Naphthol AS-D Chloroacetate esterase staining were counted in 10 microscope visual fields for one slide of each animal of the three (**B**). Renal tissues from diabetic and controls with and without TPEN treatment were subject to Western blotting assay for ICAM-1 (**C**). Data are presented as the mean ± SD (*n* = 5 at least for each group). a, *P* < 0.05 *versus* Control or TPEN group; b, *P* < 0.05 *versus* DM group. DM: diabetes.

Naphthol AS-D Chloroacetate esterase staining indicated the inflammatory cell infiltration in the glomerular and tubulointerstitial areas (Fig. 7A). Semi-quantitative analysis of the inflammatory cell infiltration showed that Zn deficiency in normal mice increased renal inflammatory cells, diabetes alone also could increase renal inflammatory cells, but Zn deficiency significantly enhanced the infiltration of inflammatory cells in the diabetic kidney (Fig. 7B). At the same time, Zn deficiency-enhanced diabetic inflammation in the kidney was confirmed by increased renal expression of ICAM-1 (Fig. 7C).

**Fig. 8:**
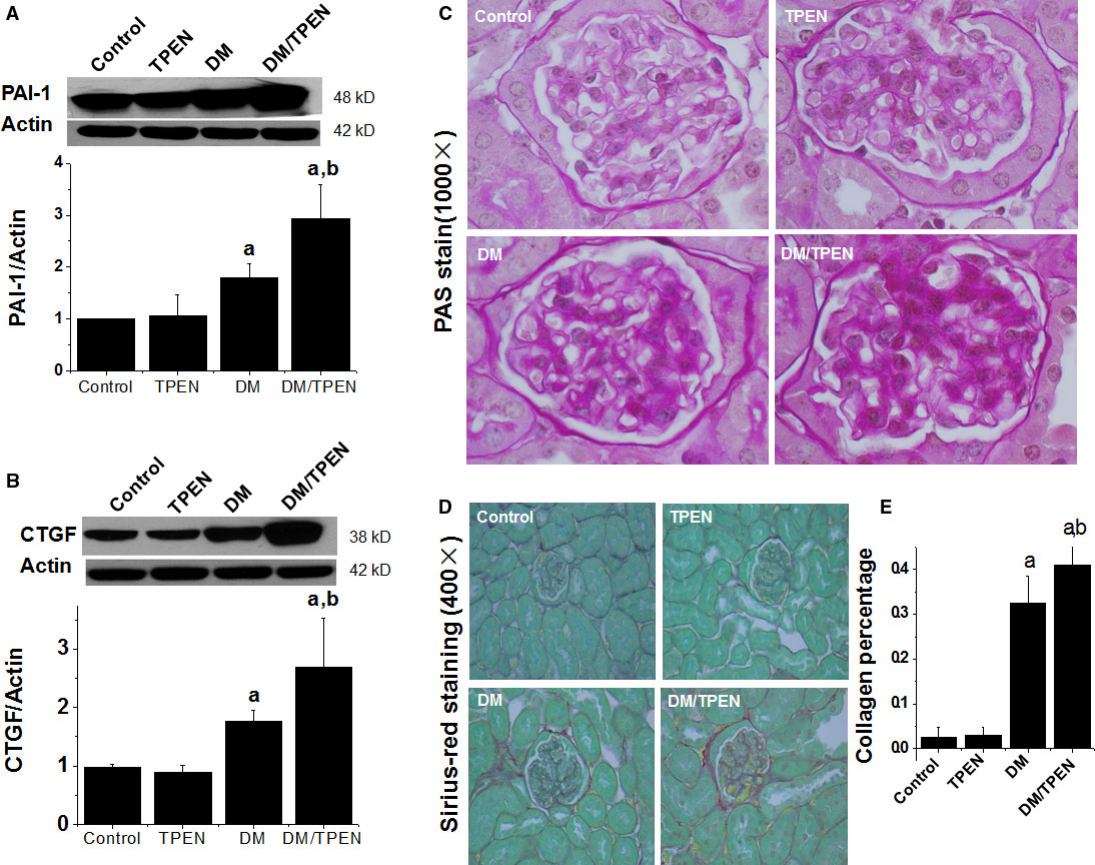
Effect of Zn deficiency on diabetes-induced renal fibrosis and histopathological changes. Renal tissues were examined by Western blotting for PAI-1 (**A**) and connective tissue growth factor (**B**) expression and by PAS (**C**) and Sirius red (**D**) staining for fibrotic response. Semi-quantitative analysis for Sirius red staining was done by computer imaging system (**E**). Data are presented as the mean ± SD (*n* = 5 at least for each group). a, *P* < 0.05 *versus* Control or TPEN group; b, *P* < 0.05 *versus* DM group. DM: diabetes.

Both oxidative stress and inflammation play important roles in initiating diabetic fibrotic effects. PAI-1 acts as both inflammatory and pro-fibrotic mediator; therefore, we measured renal PAI-1 expression, which was significantly increased in diabetic mice and further enhanced by Zn deficiency (Fig. 8A). The increased PAI-1 expression suggests the possible existence of renal fibrosis, which was confirmed by increased renal expression of CTGF, an important pro-fibrotic mediator [40]. Diabetes significantly induced renal CTGF expression, an effect enhanced by Zn deficiency (Fig. 8B).

Immunofluorescent detection of collagen IV further confirmed that diabetes increased renal accumulation of collagen IV in the glomerular area, and this effect was significantly enhanced by Zn deficiency (Fig. S6). Renal histopathological examination by PAS staining for the structure containing a high proportion of carbohydrate macromolecules (glycogen, glycoprotein, proteoglycans) demonstrated that compared to the control or TPEN group, diabetes and diabetes/TPEN groups displayed obvious mesangial cell proliferation, mesangial matrix expansion, and increased glomerular basement membrane thickness and capillary collapse (Fig. 8C). Sirius-red staining for collagen showed a significant renal collagen accumulation (Fig. 8D) followed by a semi-quantitative analysis (Fig. 8E), both which showed that Zn deficiency significantly enhanced diabetes-induced renal fibrosis.

## Discussion

Zn is an essential component of numerous proteins involved in the defense against oxidative stress [1,15,16]. Previous studies showed the induction of Nrf2 up-regulation by Zn along with a protection against oxidative damage in *in vitro* and *in vivo* systems [15,16]. For instance, Zn significantly increased glutathione (GSH) levels in the cultured retinal pigment epithelial cells through activation of the Nrf2 pathway, but the induction of GSH synthesis by Zn was inhibited by knockdown of Nrf2 expression using siRNA technology [16]. McMahon *et al*. also confirmed the induction of Nrf2 expression in the mouse embryonic fibroblasts by Zn at 100 and 300 μM [41]. Consistent with these studies, we demonstrated that exposure of human renal tubular cells to 50 μM Zn for 6–48 hrs induced Nrf2 expression and nuclear translocation. Zn-induced up-regulation of Nrf2 and its downstream antioxidant such as HO-1 was accompanied by a significant prevention of renal tubular expression of pro-fibrotic mediators CTGF and PAI-1 in response to HG/Pal, an *in vitro* diabetes condition. By depletion or supplement of Zn in the cultured cells, we found the requirement of Zn for maintaining Nrf2 expression and its transcription function in response to either HG/Pal or SFN. Our finding is in the line of a most recent study using neuronal cells, showing that nuclear translocation of Nrf2 was impaired in Zn deficient cells [42].

However, the mechanism by which Zn affects Nrf2 expression and transcription function remains largely unknown. Here, we provided the evidence for the first time that Zn stimulates the Nrf2 expression and transcription, likely by activation of Akt-dependent inhibition of Fyn nuclear translocation. First, reduction in cellular free Zn from cultured medium by TPEN decreased Nrf2 expression and nuclear accumulation. When TPEN-treated cells were supplied with additional Zn, the Nrf2 expression and nuclear translocation were restored. Second, Zn supplement up-regulates Akt function that inactivates GSK-3β by increasing its phosphorylation, leading to a significant reduction in Fyn nuclear translocation. Last, the inhibition of Akt phosphorylation by its inhibitor abolished the stimulation of Nrf2 expression and transcription by Zn. We know that Fyn, through nuclear translocation, can lead to Nrf2 exporting to the cytosol where Nrf2 is bound by Keap1 and degraded [39,43]. Fyn nuclear translocation may be prevented by activation of Akt with Zn, resulting in Nrf2 accumulation in the nuclei to transcriptionally increase multiple antioxidants, as shown in Figure 5. Our study may provide the mechanistic explanation of the recent study [21], in which rats were control-fed, alcohol-fed and alcohol-fed with Zn supplementation. They found that the alveolar macrophages isolated from alcohol-fed mice showed a significant decrease in the nuclear banding of Nrf2 compared to that from control-fed mice; however, the decreased nuclear banding of Nrf2 could be restored by adding Zn. That is probably because alcohol feeding reduces the intracellular free Zn level that stimulates Fyn into nuclear to export Nrf2 to cytosolic degradation while Zn supplement might stimulate Akt and GSK-3β phosphorylation that inhibits Fyn nuclear translocation, preventing Fyn-mediated export of Nrf2 from nuclear that restores Nrf2 nuclear binding.

Both diabetes and Zn deficiency are global health problems [22,44–47]. Diabetic patients often suffer from Zn deficiency at the late stage, particularly in the patients whose glucose was poorly controlled [48–51]. In the present research, an animal model was used to demonstrate for the first time that Zn deficiency significantly exacerbates diabetes-induced renal oxidative damage, inflammation and fibrotic effect, which was associated with down-regulation of Nrf2 expression and transcription function. Therefore, another important finding here is that Zn deficiency exacerbates diabetes-induced pathogenic changes, which was associated with the down-regulation Nrf2 expression in the animal model. As an adaptive mechanism, Nrf2 is quickly up-regulated in cells and tissues in response to various oxidative stresses, but down-regulated in cells or tissue exposed to chronic oxidative stress [19,20]. For instance, we recently found the increased expression of renal Nrf2 and its downstream antioxidants at 3-month diabetic mice, but no change or a significantly decreased expression of Nrf2 and its downstream antioxidants at 6-month diabetic mice [36]. In this study, renal Nrf2 expression remained slight increase in diabetic mice at 4 months after diabetes onset, probably because the duration of diabetes is still relatively short. However, diabetic mice with Zn deficiency showed a significant down-regulation of Nrf2 and its effector HO-1. More importantly, Zn deficiency down-regulated Nrf2 expression resulted in a significant increase in oxidative and nitrosative damage, renal inflammation and fibrosis. There was a piece of indirect evidence that supports our notion. Alcohol-fed rats had a fivefold decrease in lung bacterial clearance along with a decreased nuclear binding of Nrf2 and increased oxidative stress and damage in alveolar macrophages, compared to control-fed rats [21]. This inhibition of Nrf2 nuclear binding by alcohol feeding may be because of the induction of Zn deficiency by alcohol feeding, a common phenomenon in chronic alcohol consumption [33,52]. In support of this assumption, these effects of alcohol feeding were completely prevented by dietary Zn supplementation of these alcohol-fed rats without complete restoration of Nrf2 nuclear banding [21]. A recent study demonstrated that Zn deficiency down-regulates γ-glutamyl cysteine synthetase, the first enzyme in the GSH synthetic pathway, *via* impairment of Nrf2 nuclear translocation [42]. All these findings indirectly support the essentiality of Zn for Nrf2′s normal function. In tumour cells, Zn at sub-cytotoxic concentrations (50–100 μM) induced HO-1 expression in the MDA-MB-231 (human breast cancer) and A2780 (human ovarian cancer) cell lines in a concentration- and time-dependent manner. The induction of HO-1, as one of downstream genes of Nrf2, by Zn was detected after 4–6 hrs of treatment, reached maximal level at 8 hrs, and declined thereafter. Zn also increased Nrf2 protein expression. All these features were confirmed by our *in vitro* study. Knockdown of Nrf2 expression compromised the Zn-induced increase in HO-1 gene transcription. In addition, this study showed that the Zn-induced HO-1 gene transcription can be enhanced by clioquinol, a Zn ionophore, and reversed by pre-treatment with TPEN, as what we found in this study [53].

In summary, by systemic investigation using human renal tubule HK11 cells, we have defined the essential role of Zn in maintaining Nrf2 normal expression and transcription function. The underlying mechanism for the essential role of Zn for Nrf2 is Akt/GSK-3β-mediated inhibition of Nrf2 nuclear exporter Fyn. We further demonstrated here for the first time that Zn deficiency significantly enhanced HG/Pal-induced HK11 cell pro-fibrotic response and diabetes-induced renal oxidative damage, inflammation and fibrotic effect, likely through down-regulation of Nrf2 expression and transcription function. Considering that patients with diabetes often have certain levels of Zn deficiency predominantly because of the increased urinary Zn excretion and the restricted certain food intake [44,45], we would like to draw the attention of the patients with diabetes that proper intake of Zn may be important for the prevention of their diabetic complications, including diabetic nephropathy.

## Supporting information

**Figure S1** Nrf2 expression and translocation under different conditions. **Figure S2** Dose effects of Zn on Nrf2 expression and translocation. **Figure S3** Nrf2 translocation. **Figure S4** Effect of sulforaphane on Nrf2 and NQO1 expression of the cultured human renal tubular cells. **Figure S5** Effects of chronic TPEN treatment on blood glucose levels, the ratio of kidney weight to bw, and renal zinc level. **Figure S6** Effects of Zn deficiency on diabetes-induced renal fibrosis.
