# Supplementary material for: Zinc is essential for the transcription function of Nrf2 in human renal tubule cells in vitro and mouse kidney in vivo under the diabetic condition
Source: J Cell Mol Med. 2014 Mar 6;18(5):895–906. doi: 10.1111/jcmm.12239 (PMC4119395; doi:10.1111/jcmm.12239)
Supplement: Supplementary file 1 [file jcmm0018-0895-SD1.doc]

**Zinc is essential for the transcription function of Nrf2 in human renal tubule cells in vitro and mouse kidney in vivo under the diabetic condition**

Bing Li ^1,2,3^, Wenpeng Cui ^1,2^, Yi Tan ^2,4^, Ping Luo ^1^, Qiang Chen ^2^, Chi Zhang ^2,4^, Wei Qu ^5^, Lining Miao ^1,†^, Lu Cai ^2,4,†^

**Supplemental figures**

**Supplemental Fig. 1. Nrf2 expression and translocation under different conditions.** Human renal tubular HK 11 cells cultured on chamber slides are treated with different conditions as indicated. HG (27.5 mM) for 48 h, palmitate (P, 300 µM) for the last 6 h, TPEN (4 µM), Zn (50 µM) for the last 30 h. Immunofluorescence staining of Nrf2 is detected by fluorescent microscope (400×).

**Supplemental Fig. 2. Dose-effects of Zn on Nrf2 expression and translocation.** Human renal tubular HK 11 cells were cultured on chamber slides with treatments of HG (27.5 mM) for 48 h, palmitate (300 µM) for the last 6 h, and TPEN (4 µM) were treated with Zn at different concentrations (0, 25, 50, 75 and 100 µM) or without any treatment (Control). Immunofluorescence staining of Nrf2 is detected by fluorescent microscope (400×).

**Supplemental Fig. 3. Nrf2 translocation.** Human renal tubular HK 11 cells cultured on chamber slides were treated by HG (27.5 mM) for 48 h, palmitate (P, 300 µM) for the last 6 h and TPEN (4 µM) without (A) or with (B) Zn (50 μM). Immunofluorescence staining of Nrf2 is detected by fluorescent microscope (oil lens).

**Supplemental Fig. 4**. Effect of sulforaphane on Nrf2 and NQO1 expression of the cultured human renal tubular cells. Human renal tubular HK11 cells were treated with HG/Pal (G, 27.5 mM glucose for 48 h + 300 µM Pal for the last 6 h) with or without sulforaphane (S, 3μM for 48h), TPEN (T, 8 µM) or Zn (Z, 50 µM) for the last 30 h. Then the cells were subject to Western blotting assay for the expression of Nrf2 and NQO1. Experiments were repeated at least three times and the data are presented as mean ± SD. a, P< 0.05 vs. G group; b, P<0.05 vs. S/G/T group; c, P<0.05 vs. S/G group. G: HG/Pal; T: TPEN; Z: Zn; S: sulforaphane.

**Fig. 5.** **Effects of chronic TPEN treatment on blood glucose levels, the ratio of kidney weight to body weight, and renal zinc level.** Diabetic mice induced with MLD-STZ and age-matched control mice were given TPEN at 5 mg/kg every day for four months, and then the blood glucose levels (A), the ratio of kidney weight to body weight (B), and the renal Zn level (C) were examined. Data are presented as mean ± SD (n = 5 at least in each group). a, P <0.05 vs. control group; b, P<0.05 vs. DM group. DM: diabetes.

**Supplemental Fig.6. Effects of Zn deficiency on diabetes-induced renal fibrosis.** Renal fibrosis was examined by immunofluorescent staining of Collagen IV. Semi-quantitative analysis was done by computer imaging system. a, P< 0.05 vs. Control or TPEN group; b, P<0.05 vs. DM group. DM: diabetes.
